# Is ligation of hernial sac during orchiopexy mandatory?

**DOI:** 10.4103/0971-9261.55155

**Published:** 2009

**Authors:** Veena Kumari, Nilay Biswas, Nilanjan Mitra, Hiralal Konar, Dipak Ghosh, Sukanta K. Das

**Affiliations:** Department of Pediatric Surgery, Medical College and Hospital, Kolkata, West Bengal, India

**Keywords:** Hernia Inguinal, orchiopexy, undescended testis

## Abstract

**Aim:**

Traditionally, ligation of hernial sac during orchiopexy is considered mandatory to prevent postoperative development of hernia. A prospective study was carried out to see if it is actually required based on the fact that any peritoneal defect closes within 24 hours by metamorphosis of the in situ mesodermal cells.

**Methods:**

Fifty cases of undescended testis, age ranging from eight months to 12 years were enrolled. All of them underwent standard orchiopexy without ligation of the hernial sac.

**Results:**

Follow up of all cases ranged between 1.5 years to three years. Not a single case was reported with evidence of hernia.

**Conclusions:**

It is unnecessary to ligate the hernial sac during orchiopexy.

## INTRODUCTION

The conventional and accepted technique of orchiopexy recommends that ligation of the hernial sac is mandatory for prevention of postoperative development of hernia. However, it has been seen that during laparoscopic orchiopexy there is no difference to simple suturing when peritoneum is incised, hernia sac dissected and left alone. This may be due to the fact that any peritoneal defect closes within 24 hours by metamorphosis of the *in situ* mesodermal cells. We report the results of a study on nonligation of hernia sac during conventional orchiopexy.

## MATERIALS AND METHODS

This prospective study was conducted between April 2004 and 2007. Fifty children with an age range of eight months to 12 years with a diagnosis of undescended testis were included. Of the 50 cases, 38 were unilateral and 12 bilateral cases. Of the 38 unilateral undescended testis, 22 were right-sided and 16 left-sided. In all the cases, testis was palpable. Clinically, no cases presented with hernia. After the baseline investigations, informed consent of the parents was taken and the procedure explained to those parents who were able to understand. All children underwent standard orchiopexy without the ligation of the hernia sac. No special investigative workup was done.

The hernia sac was dealt with after complete mobilization of the testis through an inguinal incision. The sac was first opened up, divided and the proximal end of the divided sac was very gently peeled off with dissecting forceps as high as possible without damaging the cord structures. It was left as such without ligation. Standard orchiopexy was then performed by making subdartos pouch.

## RESULTS

All the patients were followed up for 1.5 years to three years. No inguinal hernia was detected during the regular follow-up in any child.

## DISCUSSION

Hernia sac has been routinely dissected and meticulously freed from the cord structures and suture ligated proximally during inguinal orchiopexy. This is done to achieve adequate length of the cord to bring down the testes to the scrotum to its normal position and prevent development hernia postoperatively.

In cases of inguinal hernia in children, Mohta *et al*.[1] suggested that nonligation of hernia sac during herniotomy in children has no untoward effect on the early complications and recurrence rate. The study was based on the fact that peritoneal defect closes by metamorphosis of the *in situ* mesodermal cells. Earlier, Shulman *et al*.[2] showed that ligation of hernia sac in adult herniorrhaphy is a needless step. A prospective study of laparoscopic inguinal hernia repair in children by Schier[3] showed that there is no difference to simple suturing when peritoneum was incised and hernia sac resected. He stressed that an open internal inguinal ring is not an inguinal hernia.[4] During laparoscopic orciopexy, Handa *et al*.[5] showed that closure of the internal ring is not necessary. Mobilization of undescended testes leaves a raw surface which coupled with the presence of the pulled through spermatic cord results in effective closure of the internal inguinal ring.

In this study we did not ligate the hernia sac during inguinal orchiopexy. After dissecting the hernia sac free from the cord, we simply gently peel off the proximal cut end of the hernia sac as high as possible. We have performed 50 cases of inguinal orchiopexy with this procedure and followed up for 1.5 to three years. We did not find any complication or untoward effect. So we conclude that ligation of hernia sac is not necessary in inguinal orhiopexy. We found a few other advantages also: Time saving: Several minutes of operating time are saved as we can avoid the holding of the proximal cut end of the hernial sac with multiple small haemostatic forceps and suture ligating it, especially when the sac is very thin and tends to tear very easily.

Length of testicular vessel: It is found that the most important criteria for bringing down the testes in the scrotum is the length of the testicular vessels; in our procedure extra length of the testicular vessel can be achieved by peeling off the peritoneum as high as possible. Accidental ligation of the cord structures is avoided.

Our experience suggests that routine ligation of the hernial sac is not mandatory. This reduces the operative time in all cases and eliminates the possibility of accidental ligation of cord structures.
